# TLR4 Signaling in MPP^+^-Induced Activation of BV-2 Cells

**DOI:** 10.1155/2016/5076740

**Published:** 2016-01-05

**Authors:** Peng Zhou, Ruihui Weng, Zhaoyu Chen, Rui Wang, Jing Zou, Xu Liu, Jinchi Liao, Yanping Wang, Ying Xia, Qing Wang

**Affiliations:** ^1^Department of Neurology, The Third Affiliated Hospital of Sun Yat-Sen University, 600 Tianhe Road, Guangzhou, Guangdong 510630, China; ^2^Department of Neurology, The Second Affiliated Hospital of Guangzhou Medical University, 250 Changgang Dong Road, Guangzhou 510260, China; ^3^Department of Neurosurgery, The University of Texas Medical School at Houston, Houston, TX 77030, USA

## Abstract

*Aims.* This work was conducted to establish an* in vitro* Parkinson's disease (PD) model by exposing BV-2 cells to 1-methyl-4-phenylpyridinium (MPP^+^) and exploring the roles of TLR2/TLR4/TLR9 in inflammatory responses to MPP^+^.* Methods/Results.* MTT assay showed that cell viability of BV-2 cells was 84.78 ± 0.86% and 81.18 ± 0.99% of the control after incubation with 0.1 mM MPP^+^ for 12 hours and 24 hours, respectively. Viability was not significantly different from the control group. With immunofluorescence technique, we found that MPP^+^ incubation at 0.1 mM for 12 hours was the best condition to activate BV-2 cells. In this condition, the levels of TNF-*α*, IL-1*β*, and iNOS protein were statistically increased compared to the control according to ELISA tests. Real time RT-PCR and western blot measurements showed that* TLR4* was statistically increased after 0.1 mM MPP^+^ incubation for 12 hours. Furthermore, after siRNA interference of* TLR4* mRNA, NF-*κ*B activation and the levels of TNF-*α*, IL-1*β*, and iNOS were all statistically decreased in this cell model.* Conclusion.* MPP^+^ incubation at the concentration of 0.1 mM for 12 hours is the best condition to activate BV-2 cells for mimicking PD inflammation in BV-2 cells. TLR4 signalling plays a critical role in the activation of BV-2 cells and the induction of inflammation in this cell model.

## 1. Introduction

Parkinson's disease (PD) is characterized by the loss of dopaminergic neurons in the substantia nigra (SN) and is the most common age-related neurodegenerative disease. Affecting roughly 2% of elderly people (≥65 years old), PD includes symptoms such as muscle rigidity, bradykinesia, and disabling motor abnormalities such as tremors and postural instability [1–3]. Although PD has been heavily researched over the last two decades, the precise etiology of the disease is still unknown. However, research in recent years has provided substantial evidence supporting the hypothesis that oxidative stress and inflammation both likely play a major role in disease pathogenesis [4]. Since then, there have been numerous reports that suggest that neuroinflammatory processes play a critical role in PD pathogenesis. In addition to the presence of activated microglia, increased levels of proinflammatory cytokines including TNF-*α* and IL-1*β* and enzymes associated with inflammation such as inducible nitric oxide synthase (iNOS) have been observed in the Parkinsonian brain [5]. In fact, cytokines released by microglia contribute to the elimination of pathogens and also act as second messengers that activate NF-*κ*B signaling, which results in further expression of proinflammatory cytokines [6].

Microglia are inhabitant immune cells of the brain that execute principle inflammatory feedback under various neurodegenerative conditions in the brain. In the event of brain damage or infection, microglia become activated and secrete a variety of proinflammatory mediators and other potentially neurotoxic factors which can have deleterious effects on neighboring neurons [7]. Suppression of microglia activation has been suggested as a possible therapeutic intervention that may alleviate the progression of various neurodegenerative diseases including PD. A BV-2 cell line established by Blasi et al. [8] displayed microglia-like characteristics and has been intensively studied in order to elucidate the mechanisms of microglia-like activation. Toll-like receptors (TLRs), such as TLR2/TLR4/TLR9, are expressed by microglia and enable them to sense that peripheral nerve injury has occurred [9–11]. Activation of TLR2/TLR4/TLR9 results in proinflammation via MyD88 dependent signaling. However, it is now recognized that TLR2/TLR4/TLR9 are less specific, as their signaling is also activated by substances released by stressed and damaged cells. Activation of TLR2/TLR4/TLR9 signaling, whether by components of invading pathogens or by endogenous danger signals, leads to the production and release of proinflammatory cytokines [12], which are intimately linked to the glial contribution to neuropathic pain [13]. At present, it is unclear which TLR signal (TLR2/TLR4/TLR9) plays the most important role in microglia activation in response to environmental stress that leads to PD pathophysiology. In MPTP-treated animal models and various cell models of PD, the expression of TLRs was increased significantly compared to the control model. This increase is also related to microglia activation. Whether or not MPP^+^, the derivative of MPTP, participates in the process of microglia activation is unknown, as is the mechanism of this activation process.

We conducted this study to explore this fundamental issue in BV-2 microglia-like cells. Since the neurotoxin 1-methyl-4-phenylpyridinium (MPP^+^) causes oxidative stress and mitochondrial dysfunction and thus injures dopaminergic neurons, leading to PD pathophysiology [14–16], we applied MPP^+^ to BV-2 cells to induce PD-like pathophysiology in the* in vitro* model and detected the changes in TLR2/TLR4/TLR9 expression and inflammatory reaction. Moreover, we explored the role of TLR2/TLR4/TLR9 in the inflammation reaction and the probable signaling pathway in this process.

## 2. Materials and Methods

### 2.1. Reagents

Dulbecco's modified Eagle's medium (DMEM), penicillin-streptomycin solutions, and fetal bovine serum (FBS) were purchased from Gibco (Grand Island, NY, USA); 1-methyl-4-phenylpyridinium (MPP^+^) and 3-(4,5-dimethyl-2-thiazolyl)-2,5-diphenyl-2H-tetrazolium bromide (MTT) were obtained from Sigma (St. Louis, MO, USA). Anti-TLR2 antibodies were obtained from Millipore (Billerica, MA, USA). Anti-TLR4 and anti-TLR9 antibody and Cy3-labeled goat anti-rabbit IgG (H + L) were purchased from Abcam (Cambridge, UK); tumor necrosis factor- (TNF-) *α*, interleukin- (IL-) 1*β*, and iNOS enzyme-linked immunosorbent assay (ELISA) kit were purchased from Nanjing KeyGen Biotech (Nanjing, China); reagents for qPCR were purchased from Takara Bio (Dalian, China).

### 2.2. Cell Culture

The BV-2 cell line was obtained from cell bank of Sun Yat-Sen University. The BV-2 cell line was grown and maintained in DMEM supplemented with 10% heat-inactivated fetal bovine serum (FBS), 100 U/mL penicillin, and 100 *μ*g/mL streptomycin at 37°C in a humidified incubator under 5% CO_2_.

### 2.3. Assessment of Cell Viability

Cell viability was determined by measuring the reduction of MTT to formazan. BV-2 cells seeded at 2.5 × 10^4^ cells/mL in a 96-well plate were pretreated with various doses of MPP^+^ (0.1 mM, 0.3 mM, 0.5 mM, and 1 mM) for 12 h and 24 h or with 0.1 mM concentration of MPP^+^ at various time intervals (1 h, 3 h, 6 h, 12 h, and 24 h). Results are representative of three individual experiments. According to the results of cell viability by MPP^+^, the appropriate concentration and time, respectively, are 0.1 mM and 12 h or 24 h and the same conditions were used in further experiments.

### 2.4. Cytokine Assay

BV-2 cells seeded at 2.5 × 10^4^ cells/mL in a 24-well plate were pretreated with various doses of MPP^+^ (0.1 mM, 0.3 mM, 0.5 mM, and 1 mM) for 12 h. In addition, BV-2 cells were transfected with TLR4 siRNA and then pretreated with 0.1 mM MPP^+^. The culture supernatants were collected. The cytokines in BV-2 cell culture supernatants were determined by ELISA kit. Supernatants from BV-2 cells were tested for the secretion of TNF-*α*, IL-1*β*, and iNOS. ELISAs were performed according to the manufacturer's instructions.

### 2.5. Real Time RT-PCR

BV-2 cells were pretreated with 0.1 mM MPP^+^ for 12 h. Control and MPP^+^ cells were collected, and total RNA was extracted with TRI reagent. Total RNA (1 *μ*g) was then reverse-transcribed in a reaction mixture containing 1 U RNase inhibitor, 500 ng random primers, 3 mM MgCl_2_, 0.5 mM dNTP, and 10 U reverse transcriptase in RT buffer. The sequences of the synthetic oligonucleotides were as follows: TLR2 (sense: 5′ TCT AAA GTC GAT CCG CGA CAT 3′; antisense: 5′CTA CGG GCA GTG GTG AAA ACT), TLR4 (sense: 5′GCC TTT CAG GGA ATT AAG CTCC 3; antisense: 5′ GAT CAA CCG ATG GAC GTG TAA A 3′), TLR9 (sense: 5′ ATG GTT CTC CGT CGA AGG ACT3′; antisense: 5′ GAG GCT TCA GCT CAC AGG G3′), and *β*-actin (sense: 5′ GGC TGT ATT CCC CTC CAT CG′; antisense: 5′ CCA GTT GGT AAC AAT GCC ATG T′). Real time RT-PCR analyses of GAPDH, TLR2, TLR4, and TLR9 were performed using the Roche universal probe library detection system. Relative quantification of gene expression was performed using the comparative threshold (CT) method. Changes in mRNA expression levels were calculated following normalization to GAPDH. The ratios obtained after normalization are expressed as fold change over corresponding controls.

### 2.6. Western Blotting Analysis

Western blotting analysis was conducted as described previously using the antibodies listed above. Protein concentrations were determined by the bicinchoninic acid protein assay. Equal amounts of protein were resolved in SDS-PAGE and transferred electrophoretically onto a nitrocellulose membrane (Merck Millipore). The membrane was blocked with 5% skimmed milk for 2 hours at room temperature and incubated overnight at 4°C with the corresponding antibodies (all diluted 1 : 1000 in primary antibody dilution buffer) and GAPDH (loading control diluted 1 : 1000 in primary antibody dilution buffer). After the membrane was washed three times for 5 minutes each time in washing buffer, it was incubated with the appropriate HRP-conjugated secondary antibody (diluted 1 : 1000 in secondary antibody dilution buffer) for 2 hours at room temperature. Immunodetection was carried out using enhanced chemiluminescence reagent according to the manufacturer's instructions (BeyoECL Plus). Equivalent protein loading and transfer efficiency were verified by staining for GAPDH.

### 2.7. Immunofluorescence

BV-2 microglia cells seeded at 2.5 × 10^4^ cells/mL on sterile cover slips were cultured in 24-well plates. BV-2 cells were pretreated with 0.1 mM MPP^+^ for 12 h. Then cells were fixed in 4% paraformaldehyde and permeabilized in 0.5% Triton X-100 for 30 min. After blocking with 5% nonfat milk in PBS buffer, cells were incubated with rabbit anti-TLR2, TLR4, and TLR9 antibodies for 1 h at room temperature. After brief wash, cells were incubated with Cy3-conjugated secondary antibody (1 : 500, Molecular Probes). Finally, the cells were washed again, mounted with VECTASHIELD hard mount with DAPI, and visualized using fluorescence microscope.

### 2.8. TLR2 and TLR4 Knockdown by siRNA Transfection

BV-2 cells were transfected with TLR2 and TLR4 siRNA using siRNA transfection reagent according to the manufacturer's protocol. The sequences targeting TLR2 and TLR4 correspond to the coding region nucleotides as follows: mouse TLR2 (A: sense, 5′GGC UGC AAG AGC UCU AUA UUU 3′; antisense, 5′ AUA UAG AGC UCU UGC AGC CUU 3′; B: sense, 5′GCU CUA UAU UUC CAG AAA UUU 3′; antisense, 5′ AUU UCU GGA AAU AUA GAG CUU 3′; C: sense, 5′TGG AAA GCA TAC CAA TTU U 3′; antisense, 5′ AAT TGT GGT ATG CTT TCC AUU 3′) and TLR4 (A: sense, 5′GCC CAU AUU UGA CUA UAA UU 3′; antisense, 5′ UUA UAG UCA AAU AUG GGC CU 3′; B: sense, 5′CCC AGU CUG UUU GCA AUU AUU 3′; antisense, 5′ UAA UUG AAG UCU AUG GAG GUU 3′; C: sense, 5′GCC CGT TAT TCT GAC AGT TUU 3′; antisense, 5′ AAC TGT CAG AAT AAC GGG CUU 3′). For negative control, BV-2 cells were transfected with the same concentration of control siRNA (sense: 5′UUC UCC GAA CGU GUC ACG UUU3′; antisense: 5′ ACG UGA CAC GUU CGG AGA A 3′). We adopted real time RT-PCR to detect the expression of TLR2 mRNA and TLR4 mRNA after interference and chose the most efficient interference sequence.

### 2.9. Electrophoretic Mobility Shift Assay (EMSA)

Oligonucleotides containing a nuclear factor- (NF-) *κ*B binding site were used as probes. Specific binding was confirmed through a competition experiment using a 50-fold excess concentration of unlabeled, identical oligonucleotide, with further steps carried out as previously reported. The protein/DNA complex was separated from the free probe in a 4.8% polyacrylamide gel in 0.5x TBE buffer (44.5 mM Tris, 44.5 mM boric acid, and 1 mM EDTA). After electrophoresis, the gel was dried and autoradiographed.

### 2.10. Statistical Analysis

The data are presented as the mean ± SE and statistical comparisons between groups were performed using one-way ANOVA followed by Student's *t*-test between two populations based on the assumption that both populations have normal distribution. A *P* value <0.05 was considered significant.

## 3. Results

### 3.1. MPP^+^-Induced Activation of BV-2 Cells and the Reduction of Their Viability

**Figure 1:**
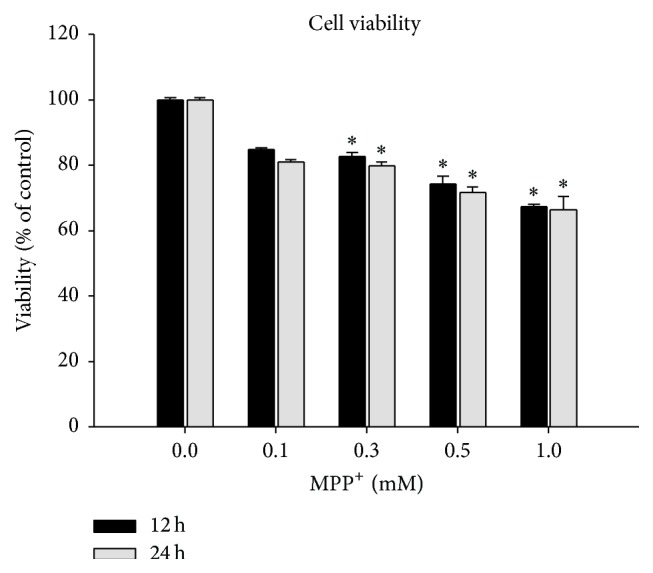
Comparisons of cell viability between control and MPP^+^-exposed BV-2 cells. MPP^+^ was applied to the BV-2 cells at four concentrations (0.1 mM, 0.3 mM, 0.5 mM, and 1 mM) and the incubation times were* 12 hours and 24 hours*. Note that there was no significant difference in cell viability between 0.1 mM MPP^+^ and control groups and there was a significant difference in cell viability between 0.3 mM MPP^+^ and the control, between 0.5 mM MPP^+^ and the control, and between 1 mM MPP^+^ and the control at 12 hours and 24 hours. ^*∗*^ *P* < 0.05.

**Figure 2:**
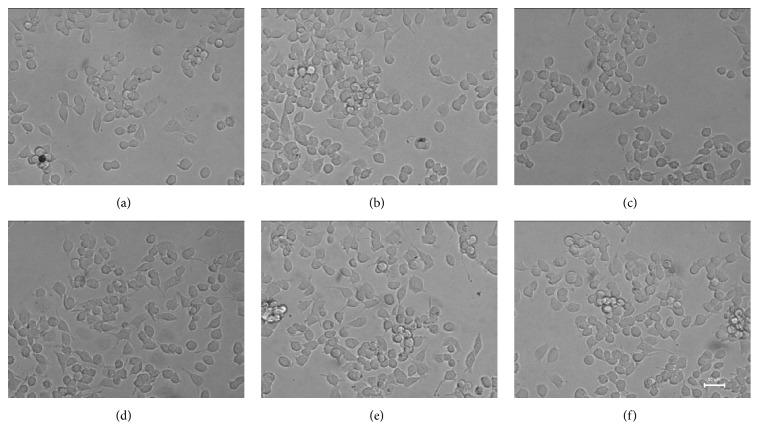
The morphology of BV-2 cells exposed to MPP^+^ at various time points (20x). (a) 0 h; (b) 1 h; (c) 3 h; (d) 6 h; (e) 12 h; (f) 24 h. Note that the activation status of BV-2 cells was most evident after incubation with 0.1 mM MPP^+^ for 12 hours.

We evaluated the effects of 12-hour and 24-hour MPP^+^ exposure on BV-2 cell viability by MTT assay.  Figure 1 shows BV-2 cell viability after 12-hour and 24-hour MPP^+^ treatment in a concentration-dependent manner. The viability of BV-2 cell was 84.78 ± 0.86% and 81.18 ± 0.99% of the control after the incubation with 0.1 mM MPP^+^ for 12 hours and 24 hours, respectively, which were not significantly different from the control group. However, the cell viability was 81.40 ± 1.86 (0.3 mM, 12 h), 74.35 ± 4.35% (0.5 mM, 12 h), 67.49 ± 0.86% (1 mM, 12 h), 79.88% ± 1.79% (0.3 mM, 24 h), 71.73% ± 2.36% (0.5 mM, 24 h), and 66.52% ± 5.50% (1 mM, 24 h), respectively, which showed significant reduction compared to the control group (Figure 1). The number of BV-2 cells increased when exposed to 0.1 mM MPP^+^ at 1 h, 3 h, 6 h, and 12 h, in a time-dependent manner. BV-2 cell bodies became bigger and became obviously swollen at 12 h when observing with a phase contrast microscope (20x), which suggested that cells were activated. However, the body volumes shrank by 24 h (Figure 2).

### 3.2. TLR4 Involvement in the Secretion of TNF-*α*, IL-1*β*, and iNOS by BV-2 Cells Exposed to MPP^+^

**Figure 3:**
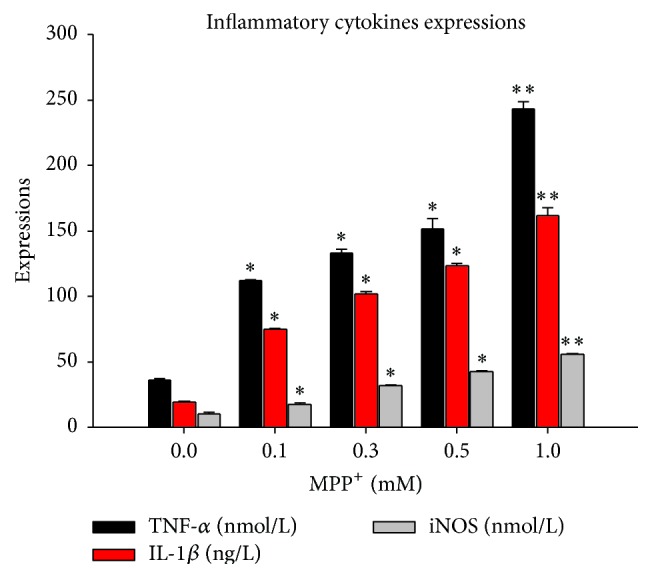
The changes in TNF-*α*, IL-1*β*, and iNOS of the BV-2 cells exposed to MPP^+^ for 12 h. MPP^+^ was applied to the cells at four concentrations (0.1 mM, 0.3 mM, 0.5 mM, and 1 mM), and the incubation time was 12 hours. Note that there were significant differences in protein expression between MPP^+^ and control groups. ^*∗*^ *P* < 0.05 versus control; ^*∗∗*^ *P* < 0.01 versus control.

**Figure 4:**
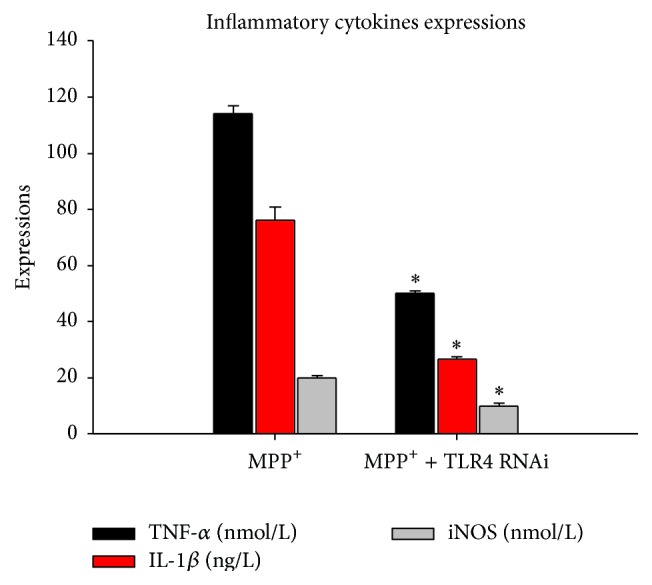
The changes of TNF-*α*, IL-1*β*, and iNOS in TLR4 siRNA treated BV-2 cells after exposure to MPP^+^ for 12 h. Note that, after the treatment with* TLR4* siRNA interference, the levels of TNF-*α*, IL-1*β*, and iNOS were statistically decreased in the 0.1 mM MPP^+^ group as compared with those of 0.1 mM MPP^+^ group without the treatment with* TLR4* siRNA interference. ^*∗*^ *P* < 0.05.

Figure 3 shows the levels of TNF-*α*, IL-1*β*, and iNOS secreted by BV-2 cells pretreated with various doses of MPP^+^ (0.1 mM, 0.3 mM, 0.5 mM, and 1 mM) for 12 hours. The secretion of TNF-*α*, IL-1*β*, and iNOS was significantly greater in the BV-2 cells exposed to 0.1 mM MPP^+^ treatment than in the control group, which also suggested that the BV-2 cells were activated. In sharp contrast, after the cells were transfected with TLR4 siRNA, the secretion of TNF-*α*, IL-1*β*, and iNOS significantly decreased in response to the same MPP^+^ treatment (Figure 4).

### 3.3. Increased* TLR4* mRNA in BV-2 Cells in Response to MPP^+^ Treatment

**Figure 5:**
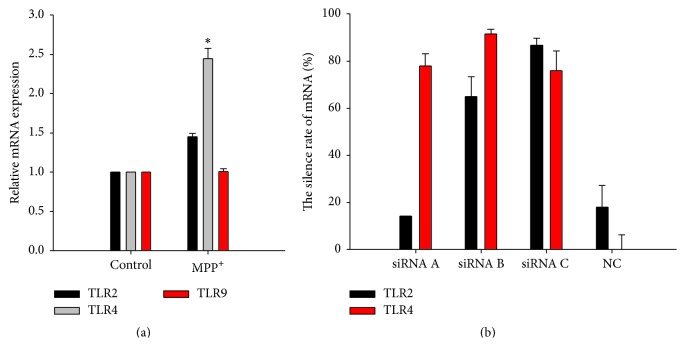
(a) The mRNA levels of TLR2, TLR4, and TLR9 in the BV-2 cells exposed to MPP^+^ for 12 h. Note that there was a statistical increase in* TLR4 mRNA* after 0.1 mM MPP^+^ incubation for 12 hours compared to control group (^*∗*^ *P* < 0.05). No significant differences in* TLR2 mRNA* and* TLR9 mRNA* levels were found between 0.1 mM MPP^+^ group and the control. (b) Relative silencing efficiency of TLR2 RNAi and TLR4 RNAi in the BV-2 cells. The mRNA was measured after the cells were incubated with 0.1 mM MPP^+^. Note that the silencing efficiency was over 70% in the group of TLR2 siRNA C and over 90% in that of TLR4 siRNA B.

The mRNA levels of TLR2, TLR4, and TLR9 were measured by real time RT-PCR analysis and are shown in Figure 5(a). The mRNA expression of TLR4 obviously increased in BV-2 cells exposed to MPP^+^ at 12 h, with statistical significance compared to the control group. In contrast, there was no significant difference in the mRNA expression of TLR2 and TLR9 between BV-2 cells exposed to MPP^+^ and those without MPP^+^ exposure. The results suggested that expression of TLR4 may increase at transcriptional levels, which may relate to the activation of BV-2 cells and the production of proinflammatory factors.

### 3.4. Increased Expression of TLR4 Protein in BV-2 Cells in Response to MPP^+^ Treatment

**Figure 6:**
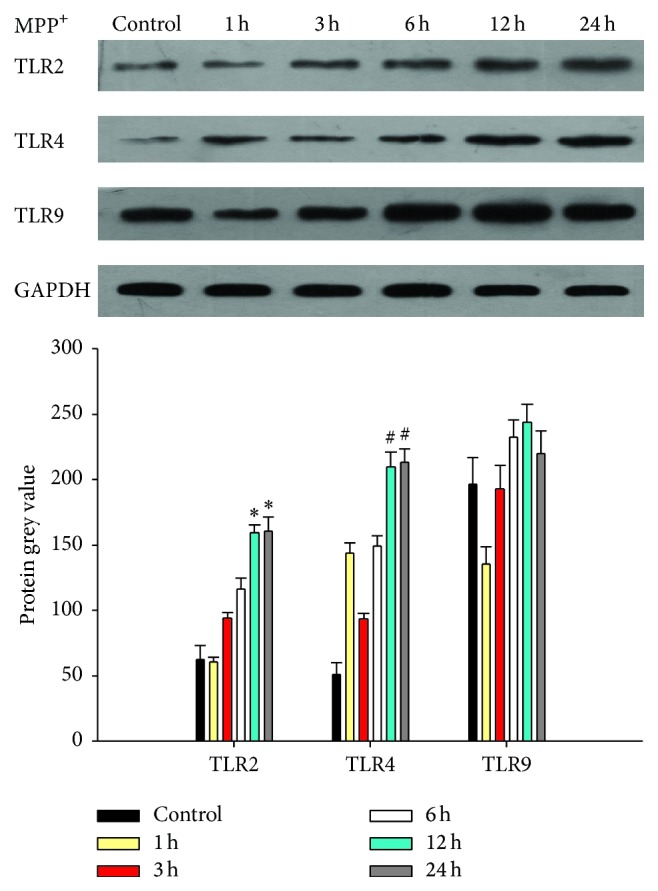
The changes in the protein levels of TLR2, TLR4, and TLR9 in the BV-2 cells exposed to MPP^+^ for different durations. Note that there was a statistical increase in the level of TLR2/TLR4 protein after 0.1 mM MPP^+^ incubation for 12 hours and 24 hours (^*∗*^ *P* < 0.05 versus TLR2 control group; ^#^ *P* < 0.05 versus TLR4 control group) and no significant difference in TLR9 protein between 0.1 mM MPP^+^ group and control.

**Figure 7:**
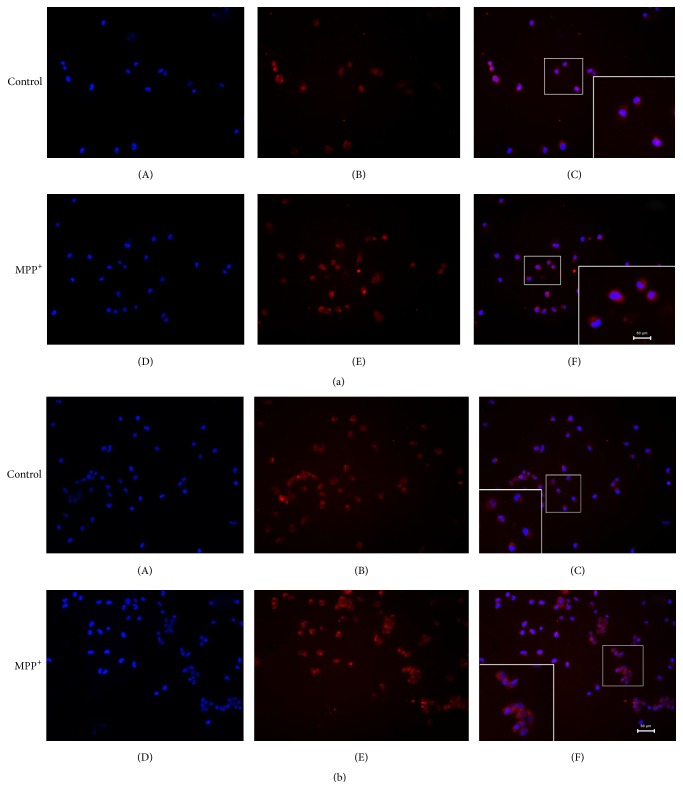
The expression of TLR2/TLR4 in BV-2 cells exposed to MPP^+^ for 12 h (fluorescent microscope image, 200x). Note that MPP^+^ treatment increased TLR4 protein in the cell membrane (a) and did not change the expression of TLR2 in the same cells (b).

The expression of TLR2, TLR4, and TLR9 protein in the BV-2 cell exposed to 0.1 mM MPP^+^ for different time durations (3, 6, 12, and 24 h) was detected by western blot and results are shown in Figure 6. The expression of TLR4 protein significantly increased at 12 h and 24 h of MPP^+^ exposure compared to the control level. These results correspond to the best time of activation. Similarly, the expression of TLR2 protein was also upregulated at 12 h and 24 h of MPP^+^ exposure. However, the increased magnitude was not significant. The expression of TLR9 protein was not significantly changed in the BV-2 cells with MPP^+^ treatment at any time point. Furthermore, we used immunofluorescence to localize the expression of TLR2 and TLR4 in the cells (Figure 7). A relatively low level of TLR4 protein was detected in the membrane of untreated BV-2 cells, while it was significantly upregulated after treatment with MPP^+^. In contrast, the expression of TLR2 did not show upregulation in the membranes of MPP^+^ treated BV-2 cells.

### 3.5. Inhibition of NF-*κ*B Activation by TLR4 RNA Interference in BV-2 Cells Exposed to MPP^+^

**Figure 8:**
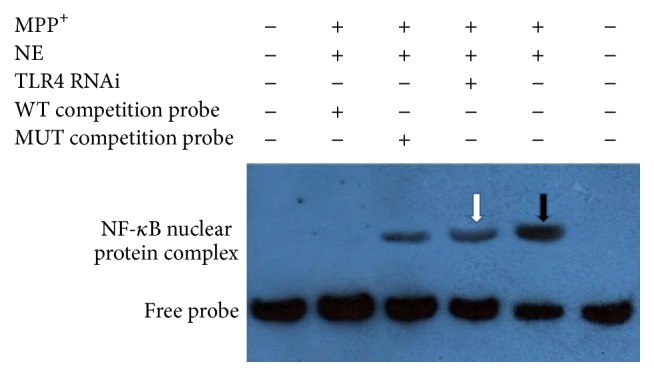
The effect of TLR4 RNA interference on NF-*κ*B activation in BV-2 cells exposed to MPP^+^ for 12 h (negative control group; WT cold probe group; MUT cold probe group; RNAi group; non-RNAi group; negative control group). Note that, in the group treated with TLR4 RNA interference, NF-*κ*B activation decreased compared to that of non-RNAi group.

In addition, we adopted real time RT-PCR to detect the expression of TLR2 mRNA and TLR4 mRNA after interference and chose the most efficient interference sequence. The results demonstrated that the TLR2 siRNA C sequence interference ratio was over 70%, shown in Figure 5(b), and TLR4 siRNA B sequence was over 90%. TLR4 siRNA B sequence was used in the next experiment. We examined the effect of TLR4 RNA interference on NF-*κ*B activation in BV-2 cells exposed to MPP^+^. EMSA data (Figure 8) showed that TLR4 RNA interference inhibited NF-*κ*B-DNA binding, suggesting that TLR4 RNAi attenuates MPP^+^-induced NF-*κ*B activation in the BV-2 cells.

## 4. Discussion

MPTP is a neurotoxin and is usually used to induce PD in* in vivo* model [15]. MPTP in the brain metabolizes into MPP^+^, and it is suggested that MPP^+^ is taken up into the neuron by the dopamine transporter (DAT) [17]. Whether MPP^+^ affects the activation of microglia directly was not reported. To determine which TLR signal pathway plays the most important role in microglial activation in response to environmental stress that leads to PD pathophysiology, we investigated, in microglia-like BV-2 cells, differential roles of TLR2, TLR4, and TLR9 in inflammatory responses of TNF-*α*, IL-1*β*, and iNOS to MPP^+^ stress and found that TLR4 plays a critical role in the inflammatory responses.

Microglia are resident macrophages in the central nervous system and are primarily responsible for the inflammatory response in the pathogenesis of various neurodegenerative diseases including PD. Multiple lines of evidence have suggested that microglial activation is involved in various neurodegenerative diseases and has been proven to be critically responsible for progressive neurodegeneration [18]. In the present study, we used the BV-2 cell line, which is a well-known murine microglia-like cell line showing similar phenotypic and functional properties to reactive microglial cells. Several papers suggested the suitability of BV-2 microglial cells as an alternative model system for primary microglial culture or for animal experiments examining neuroinflammation [19].

When BV-2 cells were pretreated with MPP^+^ (0.3 mM, 0.5 mM, and 1 mM), cell viability markedly decreased compared to untreated cells. This decrease was closely related to the production of reactive oxygen species and mitochondrial-mediated apoptotic processes [20]. Through morphology observation, we found that the cells pretreated with 0.1 mM MPP^+^ were obviously activated at 12 h. The level of cytokines TNF-*α*, IL-1*β*, and iNOS significantly increased in BV-2 cells induced by MPP^+^. The above results demonstrate that MPP^+^ incubation at 0.1 mM for 12 hours is the best condition to activate the cytokines. Overproduction of iNOS has been correlated with oxidative stress and is regulated by NF-*κ*B, which has a pivotal role in mediating the immediate, early stages of immune and inflammatory responses [21, 22]. Furthermore, NF-*κ*B is also reported to execute the generation of proinflammatory cytokines such as IL-1*β* and TNF-*α* [23], which ultimately exacerbates neurotoxicity and cognitive deficits [24, 25].

Therefore, we examined NF-*κ*B activation in BV-2 cells induced by MPP^+^ by EMSA. The results showed that NF-*κ*B was obviously activated and induced by 0.1 mM MPP^+^ at 12 h. TLR/NF-*κ*B/cytokines may also participate in BV-2 cell inflammatory response induced by MPP^+^. Further, we tested the mRNA and protein levels of TLR2, TLR4, and TLR9, which were expressed by microglia to enable them to sense peripheral nerve injury, by immunofluorescence and western blot. The expression of TLR4 protein and mRNA significantly increased compared to the control group. The expressions of TLR2 protein observed by immunofluorescence and western blot were upregulated, but not as prominently as in TLR4. It seems that TLR4/NF-*κ*B/cytokines might be obviously activated and induced by 0.1 mM MPP^+^. In order to determine the role of TLR4 in BV-2 cell inflammatory response induced by MPP^+^, TLR4 siRNA was applied. The levels of TNF-*α*, IL-1*β*, and iNOS did not increase in BV-2 cells after TLR4 RNA interference. Further experiments confirmed that TLR4 RNA interference inhibited NF-*κ*B-DNA binding. All these results suggest that TLR4 RNAi attenuates MPP^+^-induced TLR4/NF-*κ*B/cytokines activation in BV-2 cells. In the present study, MPP^+^-induced activation of TLR4/NF-*κ*B/cytokines signaling pathway and was inhibited by TLR4 RNA interference. Consistent with our data, several reports have also demonstrated increased TLR4 expression in microglial cells in response to MPTP and LPS [26–28]. A major consequence of blocking the downstream effects of TLR4 activation is probably the inhibition of production of proinflammatory cytokines.

In summary, MPP^+^ causes the inflammatory activation of BV-2 cells in a dose- and time-dependent manner with TLR4 playing a critical role in NF-*κ*B activation and proinflammatory secretion. Our results provide a new insight into TLR signaling following MPP^+^-induced inflammation in BV-2 microglia. This study especially highlights the importance of TLR4/NF-*κ*B inflammatory signaling. Inhibiting TLR4 signaling pathway might be a new protective strategy against microglial inflammation in cellular and molecular events in PD pathophysiology.

## Highlights

We conclude the following:

- MPP^+^ incubation at the concentration of 0.1 mM for 12 hours is the best condition for the establishment of an* in vitro* PD model in BV-2 cells.
- The expression of TLR4 significantly increased in the BV-2 cell exposed to MPP^+^.
- TLR4/NF-*κ*B signaling was critically involved in the activation of BV-2 cells and the induction of the inflammation in this cell model of PD.

## Abbreviations

MPP^+^: 1-Methyl-4-phenylpyridinium
PD: Parkinson's disease
TLRs: Toll-like receptors
SN: Substantia nigra
iNOS: Inducible nitric oxide synthase
DMEM: Dulbecco's modified Eagle's medium
FBS: Fetal bovine serum
TNF-*α*: Tumor necrosis factor-*α*
MTT: 3-(4,5-Dimethyl-2-thiazolyl)-2,5-diphenyl-2H-tetrazolium bromide
IL: Interleukin
CT: Comparative threshold
EMSA: Electrophoretic mobility shift assay
NF-*κ*B: Nuclear factor-*κ*B
DAT: Dopamine transporter.
